# Small Steps and Giant Leaps or Just Getting on With It?

**DOI:** 10.1093/jncics/pkab083

**Published:** 2021-10-01

**Authors:** Ian D Davis

**Affiliations:** 1 Eastern Health Clinical School, Monash University, Melbourne, Victoria, Australia; 2 Cancer Services, Eastern Health, Melbourne, Victoria, Australia; 3 Australian and New Zealand Urogenital and Prostate (ANZUP) Cancer Trials Group, Sydney, New South Wales, Australia

*20 July 1969: People around the world are glued to televisions showing grainy images of Neil Armstrong, poised on the bottom step of the* Apollo 11 *lunar module, as he utters the immortal words: “That’s probably far enough. We’ve shown we can do it. Let’s go home.”*

Research that generates information that could improve the human condition but is not properly tested in clinical trials can be likened to landing on the moon and failing to take the final one small step. Everything that has gone before—all the basic and preclinical work, the grant applications, the dogged endurance—is critical, but in a real sense it is all wasted unless the information is taken into a clinical trial so that we can discover whether outcomes are improved. Imagine how much worse it is to have the evidence and the means to improve outcomes but not to use them.

It is now more than 7 years since the CHAARTED (E3805) data were presented at the annual American Society of Clinical Oncology meeting, showing a striking improvement in overall survival with the addition of docetaxel to testosterone suppression for men with metastatic hormone-sensitive prostate cancer (1). These findings were supported by data from STAMPEDE (2). Soon afterwards, benefits of similar magnitude were observed with abiraterone (3,4), this time with no ambiguity over whether the benefit was restricted to high-volume/high-risk disease. The theme continued, with survival benefits in the same context observed with addition to testosterone suppression of apalatumide (5), enzalutamide (6-8), and again with abiraterone (9,10). Of note, all these benefits are much greater in magnitude than when the same agents are used in the castration-resistant setting. They were not the result of a lack of access to or “stealing” benefit from subsequent life-prolonging therapies. The treatments did not lead to meaningful detriments in patient-reported health-related quality of life, and, for most parts of the world, at least one of the additional therapies (docetaxel) is relatively inexpensive and has a short duration of treatment. Systemic management of hormone-sensitive prostate cancer has taken a giant leap forward for the first time in seven decades.

It would seem that all that remains is to decide which treatment to add to testosterone suppression. This choice is mainly dictated by availability and reimbursement. Some patients may be too frail or have comorbidities that mean that one or another of the available additional therapies may not be suitable for them; for those men, testosterone suppression alone may still be the best option. Such a situation might reasonably be expected to be uncommon, and certainly all guidelines advocate for the addition of one of the proven agents to testosterone suppression where clinically appropriate (11-14).

In this issue of the Journal, Wallis et al. (15) describe a situation similar to the imaginary one at the start of this editorial but taken a half-step further, analogous to the spacesuited leg waving just above the lunar dust. They report evidence from the Canadian province of Ontario indicating that the clinical community has failed to take the necessary small step. This study shows that of 3556 patients with synchronous, metastatic, hormone-sensitive prostate cancer commencing testosterone suppression in the 5 years after the CHAARTED data were presented, 78.6% were offered no additional treatment. Only about 11% received docetaxel, and a small proportion (1.5%) were inferred to have received abiraterone acetate plus prednisone. The survival of patients in this study was found to be lower than in the relevant clinical trials. The survival outcome is probably to be expected because the reported population was older overall (only those older than 65 years of age were included) and otherwise may not be the same as those included in clinical trials, although the reported Charlson comorbidity index mean scores were low.

Canada is not alone in this pattern of care and the resultant outcomes. Data from the US Veterans Health Administration showed that 62% of patients received testosterone suppression only (16). Another US study showed that 65% of patients did not receive an agent shown to improve survival in addition to testosterone suppression (17). My own country of Australia, which I would like to think is forward thinking and in favor of evidence-based treatment, unfortunately showed similar results: Only 33% of patients had docetaxel added to their treatment in 2018, although the figure was higher (64%) in patients under the age of 70 years (18). The other agents are not yet reimbursed in Australia for this indication.

What could be the explanation? It cannot be lack of awareness. The CHAARTED and STAMPEDE data for the addition of docetaxel were promoted widely. Wallis et al. (15) show that prescriptions of abiraterone increased slightly after the LATITUDE data were presented, but docetaxel prescriptions decreased and against expectations that an even higher proportion of patients overall received testosterone suppression alone. Cost and other factors affecting access might be potential considerations. Access to some of the therapies was and still is limited in many countries, but docetaxel is inexpensive and readily available, and it is still recommended even in some resource-limited countries (13,19). Wallis et al. contend that age affects the mortality of these patients, which may be true. I would propose an alternative possible explanation: perhaps age biases the treatment choices made by clinicians, and older patients sometimes may be inappropriately undertreated.

The problem may run even deeper than that. The TITAN trial of apalutamide commenced recruitment in late 2015, yet only 11% of participants received docetaxel before commencement of study therapy (5). Similarly, the ARCHES trial of enzalutamide commenced recruitment in 2016, yet only 18% of participants received docetaxel (6). These findings suggest that even those clinicians engaged in research and generating evidence were frequently unwilling to use docetaxel in many patients who were fit to receive it, despite the convincing evidence available at the time. In contrast, 45% of patients in ENZAMET received docetaxel concurrently with enzalutamide, with even higher uptake in the subgroup with high-volume disease (20). Survival outcomes in the control arm of ENZAMET were similar to those of the experimental arm of CHAARTED (8). The PEACE-1 trial (NCT01957436) was amended in 2017 to require docetaxel in the standard-of-care arms (9), and the ARASENS trial (NCT02799602, yet to be reported) used docetaxel for all participants. It is encouraging to see the evidence now being built into the design of control arms in pivotal trials.

What conclusions can we draw? We know the data. We can see the quantitative and qualitative advantages of using these therapies earlier rather than waiting for castration resistance to develop. Could it be that oncologists around the world are simply too entrenched in their ways to alter practice, even when clear and convincing evidence exists to show that we can do much better? We have been called to a “cancer moonshot” (21), but perhaps what we really need is a “groundshot” (22) that gives uniform access to treatments that are known to work. This shift will result in better care and better outcomes, not just more technology or data that are never used.

US President John F. Kennedy said in his famous speech that “we choose … to do the other things, not because they are easy, but because they are hard.” This is not hard. Changing our practice should not be a giant leap. It’s one small step for a huge benefit. Let us finally take it. Neil would expect nothing less.

## Funding

The author is employed by Monash University and Eastern Health.

## Notes

**Role of the funders:** The funders had no role in the writing of this editorial or the decision to submit it for publication.

**Disclosures:** IDD is chair of ANZUP Cancer Trials Group and global co-chair of the ENZAMET trial. Advisory boards within past 2 years (all honoraria to ANZUP): Astellas, AstraZeneca, Bayer, Eisai, Ipsen, Janssen, Merck/MSD, Pfizer, Roche. Research funding (trial funding to Eastern Health or ANZUP): Amgen, Astellas, AstraZeneca, Bayer, BMS, Eisai, Exelixis, Janssen, Medivation, Movember, MSD, Pfizer, Roche/Genentech, Seagen. Company board (unremunerated): ANZUP Cancer Trials Group.

**Author contributions:** Writing—original draft: IDD. Writing—reviewing and editing: IDD.

## Data Availability

Not applicable.

## References

[1] Sweeney CJ, Chen YH, Carducci M, et al Chemohormonal therapy in metastatic hormone-sensitive prostate cancer. N Engl J Med. 2015;373(8):737–746. 26244877 10.1056/NEJMoa1503747 PMC4562797

[2] James ND, Sydes MR, Clarke NW, et al; STAMPEDE Investigators. Addition of docetaxel, zoledronic acid, or both to first-line long-term hormone therapy in prostate cancer (STAMPEDE): survival results from an adaptive, multiarm, multistage, platform randomised controlled trial. Lancet. 2016;387(10024):1163–1177. 26719232 10.1016/S0140-6736(15)01037-5 PMC4800035

[3] James ND, de Bono JS, Spears MR, et al; STAMPEDE Investigators. Abiraterone for prostate cancer not previously treated with hormone therapy. N Engl J Med. 2017;377(4):338–351. 28578639 10.1056/NEJMoa1702900 PMC5533216

[4] Fizazi K, Tran N, Fein L, et al; LATITUDE Investigators. Abiraterone plus prednisone in metastatic, castration-sensitive prostate cancer. N Engl J Med. 2017;377(4):352–360. 28578607 10.1056/NEJMoa1704174

[5] Chi KN, Agarwal N, Bjartell A, et al; TITAN Investigators. Apalutamide for metastatic, castration-sensitive prostate cancer. N Engl J Med. 2019;381(1):13–24. 31150574 10.1056/NEJMoa1903307

[6] Armstrong AJ, Szmulewitz RZ, Petrylak DP, et al ARCHES: a randomized, phase III study of androgen deprivation therapy with enzalutamide or placebo in men with metastatic hormone-sensitive prostate cancer. J Clin Oncol. 2019;37(32):2974–2986. 31329516 10.1200/JCO.19.00799 PMC6839905

[7] Armstrong AJ, Iguchi T, Azad AA, et al Final overall survival (OS) analysis from ARCHES: A phase III, randomized, double-blind, placebo (PBO)-controlled study of enzalutamide (ENZA) + androgen deprivation therapy (ADT) in men with metastatic hormone-sensitive prostate cancer (mHSPC). Paper presented at: ESMO Congress 2021 Virtual; September 16-21, 2021. Abstract LBA25.

[8] Davis ID, Martin AJ, Stockler MR, et al Enzalutamide with standard first-line therapy in metastatic prostate cancer. N Engl J Med. 2019;381(2):121–131. 31157964 10.1056/NEJMoa1903835

[9] Fizazi K, Carles J, Foulon S, et al A phase III trial with a 2x2 factorial design in men with de novo metastatic castration-sensitive prostate cancer: overall survival with abiraterone acetate plus prednisone in PEACE-1. Paper presented at: ESMO Congress 2021 Virtual; September 16-21, 2021. Abstract LBA4_PR.

[10] Attard G, Brown L, Clarke N, et al Abiraterone acetate plus prednisolone with or without enzalutamide added to androgen deprivation therapy compared to ADT alone for men with high-risk nonmetastatic prostate cancer: primary combined analysis from two comparisons in the STAMPEDE platform protocol. Paper presented at: ESMO Congress 2021 Virtual; September 16-21, 2021. Abstract LBA4_PR.

[11] Mottet N, Cornford P, Bergh RCN, et al Prostate cancer. European Association of Urology. https://uroweb.org/guideline/prostate-cancer/. Published 2020. Accessed September 7, 2021.

[12] Virgo KS, Rumble RB, de Wit R, et al Initial management of noncastrate advanced, recurrent, or metastatic prostate cancer: ASCO guideline update. J Clin Oncol. 2021;39(11):1274–1305. 33497248 10.1200/JCO.20.03256

[13] National Comprehensive Cancer Network. Prostate cancer core resources. Version 2.2021. https://www.nccn.org/professionals/physician_gls/pdf/prostate_core.pdf. Published August 25, 2021. Accessed September 7, 2021.

[14] NHS England interim treatment options during the COVID-19 pandemic. National Institute for Health and Care Excellence Web site. https://www.nice.org.uk/guidance/ng161/resources/nhs-england-interim-treatment-options-during-the-covid19-pandemic-pdf-8715724381. Accessed September 7, 2021.

[15] Wallis CJD, Malone S, Cagiannos I, et al Real-world utilization of androgen deprivation therapy-intensification among older Canadian men with de novo metastatic prostate cancer. JNCI Cancer Spectrum. 2021. doi: 10.1093/jncics/pkab082. 10.1093/jncics/pkab082 PMC8678925 34926988

[16] Tagawa ST, Sandin R, Sah J, Mu Q, Freedland SJ. 679P Treatment patterns of metastatic castration-sensitive prostate cancer (mCSPC): a real-world evidence study. Ann Oncol. 2020;31:S541–S542.

[17] George DJ, Agarwal N, Rider JR, et al Real-world treatment patterns among patients diagnosed with metastatic castration-sensitive prostate cancer (mCSPC) in community oncology settings. J Clin Oncol. 2021;39(15 Suppl):5074–5074.

[18] Azad AA, Tran B, Davis ID, et al Predictors of real-world utilisation of docetaxel combined with androgen deprivation therapy in metastatic hormone-sensitive prostate cancer [published online ahead of print March 12, 2021]. Intern Med J. 2021. doi: 10.1111/imj.15288. 10.1111/imj.15288 33710759

[19] Chiong E, Murphy DG, Akaza H, et al; ANZUP Cancer Trials Group. Management of patients with advanced prostate cancer in the Asia Pacific region: “real-world” consideration of results from the Advanced Prostate Cancer Consensus Conference (APCCC) 2017. BJU Int. 2019;123(1):22–34. 30019467 10.1111/bju.14489

[20] Sweeney CJ, Martin AJ, Stockler MR, et al; ENZAMET Trial Investigators and the Australia and New Zealand Urogenital and Prostate Cancer Trials Group (ANZUP). Overall survival of men with metachronous metastatic hormone-sensitive prostate cancer treated with enzalutamide and androgen deprivation therapy. Eur Urol. 2021;80(3):275–279. 34030924 10.1016/j.eururo.2021.05.016

[21] Agus DB, Jaffee EM, Van Dang C. Cancer moonshot 2.0. Lancet Oncol. 2021;22(2):164–165. 33482124 10.1016/S1470-2045(21)00003-6

[22] Gyawali B, Sullivan R, Booth CM. Cancer groundshot: going global before going to the moon. Lancet Oncol. 2018;19(3):288–290. 29508746 10.1016/S1470-2045(18)30076-7

